# Supplementary material for: Water sub-diffusion in membranes for fuel cells
Source: Sci Rep. 2017 Aug 21;7:8326. doi: 10.1038/s41598-017-08746-9 (PMC5567110; doi:10.1038/s41598-017-08746-9)
Supplement: Supplementary file 1 — Electronic Supporting Information for: Water sub-diffusion in membranes for fuel cells [file 41598_2017_8746_MOESM1_ESM.pdf]

Supporting Information

Water sub-diffusion in membranes for fuel cells

Quentin Berrod[1,2], Samuel Hanot[3,4,5], Armel Guillermo[6,7,8], Stefano Mossa[6,7,8], and Sandrine Lyonnard*[6,7,8]*

*Corresponding authors

[1] Laboratoire Léon Brillouin, CEA-CNRS, CEA Saclay, 91191 Gif-sur-Yvette, France

[2] Lawrence Berkeley National Laboratory, Energy Storage Group, 94720 Berkeley, USA

[3] Institut Laue-Langevin - 71 Avenue des Martyrs - CS 20156 - 38042 Grenoble Cedex 9

[4] Unité de Bioinformatique Structurale, Institut Pasteur, Paris, France

[5] UMR 3528, Centre National de la Recherche Scientifique, Paris, France.

[6] CNRS / INAC- SYMMES, F-38000 Grenoble, France

[7] CEA / INAC- SYMMES, F-38000 Grenoble, France

[8] Université Grenoble Alpes, INAC-SYMMES, F-38000 Grenoble, France

1. Experimental section

1.1 Sorption isotherms

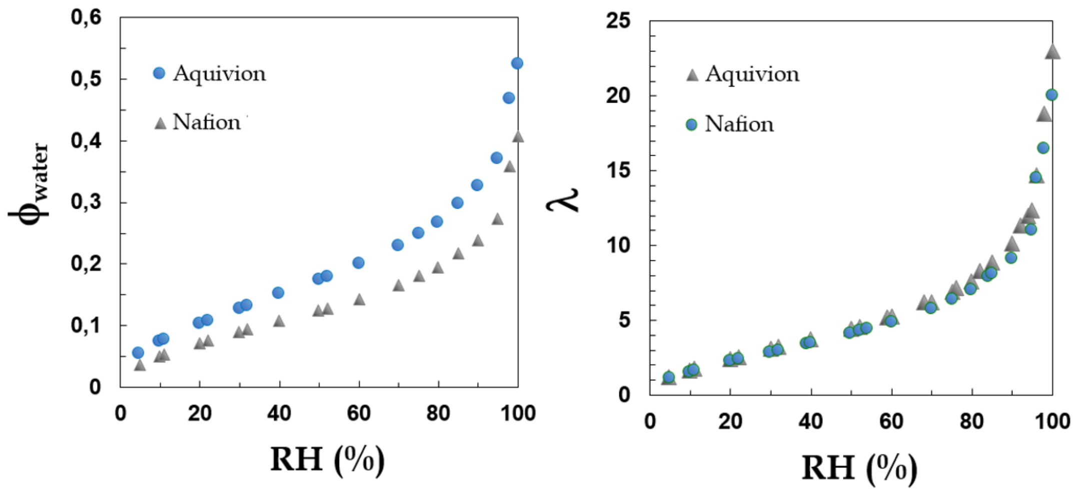

Figure S1. Sorption isotherms of Aquivion 790g/eq and Nafion 1100g/eq versus the relative humidity (RH). The water content is expressed as the water volume fraction, Φ_w (left), or the local hydration number λ, defined as the number of water molecules per sulfonic acid group (right).

2. Nanostructure by SAXS.

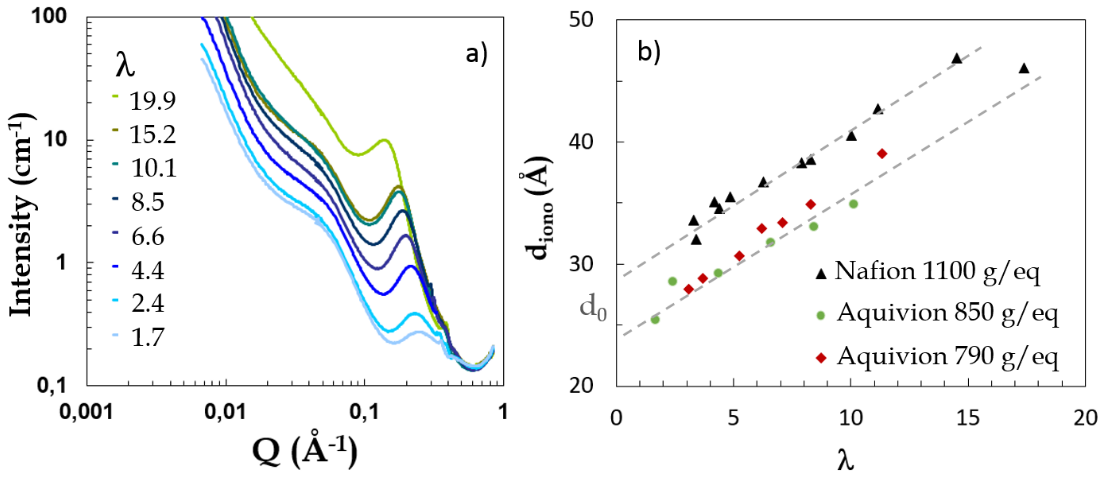

Figure S2. a) 1D SAXS spectra of Aquivion membranes as a function of the hydration number λ. b) Mean separation distance between hydrophobic aggregates, $d_{iono,}$ obtained from the ionomer peak position Q_{iono}, e.g. $d_{iono}= 2\pi/Q_{iono}$ versus λ for Nafion (1100 g/eq) and Aquivion (790 and 850 g/eq). The mean size of aggregates is obtained from the extrapolated value in dried state, d_0 = d_{iono} (λ=0). We find d_0 = 29 Å in Nafion and 24 Å in Aquivion.

3. Quasi-Elastic Neutron Scattering (QENS): data analysis and parameters.

Models with variable degree of sophistication can be used to interpret QENS data, depending on the targeted information and the complexity of the expected intertwined mechanisms. For instance, analysis based on single-Lorentzian line shapes can be used to rapidly grasp the salient features of the dynamics, or compare first-order effects in distinct systems. We have followed this approach to compare the behavior of a large set of PFSA materials, establishing some common features.[1] On the other hand, a more complete description can require: i) the collection of QENS spectra on extended timescales; ii) the simultaneous analysis of multiple data-sets using a single model over the entire Q-range; and iii) an iterative data fitting process, without undefined parameters. This program can only be realized by combining experimental data taken at different spectrometers (ToF and BS) with different resolutions.

Multi-resolution QENS experiments were therefore conducted on hydrated Aquivion membranes. Data were analyzed using the method developed in Perrin et al.,[2] based on the Gaussian model for translational diffusion.[3] Here we present a brief description of the methodology and modeling, all details can be found in the publications.

3.1 Multi-resolution QENS data analysis

Data taken on the time-of-flight and backscattering spectrometers were corrected using standard data reduction procedure as detailed by Perrin et al.,[2] including background and detector efficiency corrections. The total dynamical structure factor $S_{tot}(Q,\omega)$, ω being the energy transfer and Q the momentum transfer, of hydrated Aquivion membranes are displayed at selected values of Q in Fig. S4-S7. Together with the experimental data, we show the total theoretical function used for fitting and the various components included in the model. The experimental $S_{tot}(Q,\omega)$ obtained over an extended time-scale (instrumental resolution from 1 μeV to 100 μeV) are nicely reproduced at all hydrations and over the whole Q-range considering the existence of two populations of protons, labeled as fast (F) and slow (S) protons. The total dynamical structure factor $S_{tot}(Q,\omega)$ writes:

$$S_{tot}(Q,\omega) = A[N_F S_F(Q,\omega) + N_S S_S(Q,\omega) + P(Q)] \otimes Re(Q,\omega) + B(Q) \qquad \text{(Eq.1)}$$

where A, N_F, N_S, $S_F(Q,\omega)$, $S_S(Q,\omega)$, P(Q), Re(Q,ω) and B(Q) stand respectively for an amplitude factor containing the Debye Waller factor, the numbers of protons involved in the fast and slow dynamics, the fast and slow protons dynamical structure factors, the elastic contribution arising mostly from coherent contribution from the polymer matrix, the resolution function and a background. A phenomenological analysis of the data using two Lorentzian-shaped quasi-elastic components allow to identify the typical time-scales and nature of the molecular motions, e.g. slow protons are characterized by localized motions (Q-independent width of the slow Lorentzian) on the time-scale of τ_S ~100 ps, while fast protons have a diffusive behavior (broadening of the width of the fast Lorentzian component) on the time-scale of τ ~ few ps. Once these features established, one can proceed to a more sophisticated analysis comprising modeling of the different dynamical processes, e.g. the fast diffusive component is described using the extended Gaussian model, and the slow localized motions are described using hopping between two sites, as schematized in Fig. S3.

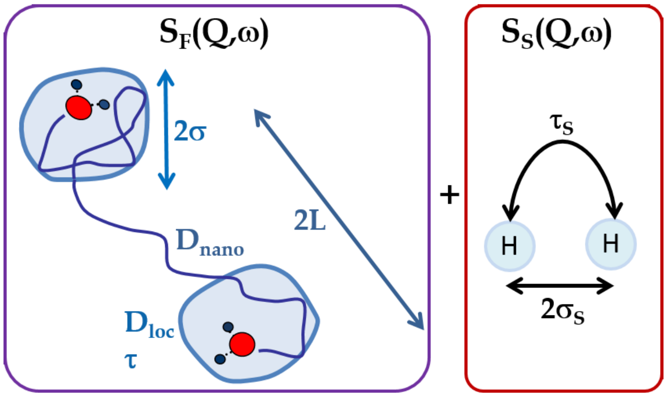

Figure S3. Schematic representation of the model used for the analysis.[3] Fast motions (left) are analysed using the extended Gaussian model, which is based on using a random Gaussian displacement variable. The species are confined in a water droplet of mean size 6σ. The intra-droplet motions are characterized by the local diffusion coefficient D_{loc} and the elementary time τ. Inter-droplet diffusion is also accounted for and quantified by the nanoscale diffusion coefficient D_{nano}. The distance between two confinement domains, 2L, is defined as $(D_{nano}/D_{loc} = exp[-L^2/(8\sigma^2)])$. The slow localized motions are analysed using a back-and-forth hopping between equivalent sites, characterized by the residence time τ_s and the jump distance σ_S. The number of fast and slow protons are labeled as N_F and N_S.

The slow protons dynamical structure factor $S_S(Q,\omega)$ is the sum of an elastic term and a Lorentzian quasielastic component:

$$S_S(Q,\omega) = A_S(\mathrm{Q})\delta(\omega) + \left(1 - A_S(\mathrm{Q})\right) \Gamma_S/(\Gamma_S{}^2 + \omega^2) \qquad \text{(Eq.2)}$$

where Γ_S stands for the Half Width at Half Maximum (HWHM) of the quasielastic component and As(Q) is the structure factor of the localized motions. The associated characteristic time τ_S is defined as $\tau_S = \hbar/\Gamma_S$.

The fast protons structure factor $S_F(Q,\omega)$ is the fourier transform of the intermediate scattering function $S_S(Q,t)$ which writes within the Gaussian model formalism:

$$I_F(Q,t) = exp\left[-Q^2\sigma^2\left(1 - exp\left(-\frac{D_{loc}t}{\sigma^2(1+2D_{loc}Q^2\tau)}\right)\right)\right] \times exp(-D_{nano}Q^2t) \qquad \text{(Eq.3)}$$

where D_{loc}, τ, σ, D_{nano}, stand for the local diffusion coefficient inside a confinement domain (so-called droplet), the characteristic time of the local jump diffusion, the confinement domain (droplet) size, and the long-range (inter-droplet) diffusion coefficient, respectively.

The data analysis using the total dynamical structure factor of Eq. 1 is performed simultaneously on the multi-resolution sets of QENS data through a back-and-forth procedure, as described in our previous study on Nafion membranes. We find a very good agreement between the experimental spectra of each hydrated Aquivion membranes and the model using a single set of parameters (N_F, N_S, D_{loc}, σ, τ, D_{nano}, τ_S and σ_s) (Fig. S4-S7). This is achieved at each instrumental resolution (1, 20, 30, 90 and 100 μeV) and over the whole Q-range, which strongly supports our interpretations and assesses the fitting procedure. Although a number of parameters have to be adjusted, they are unambiguously established given the number of constraints imposed by the extended set of data to be consistently reproduced simultaneously.

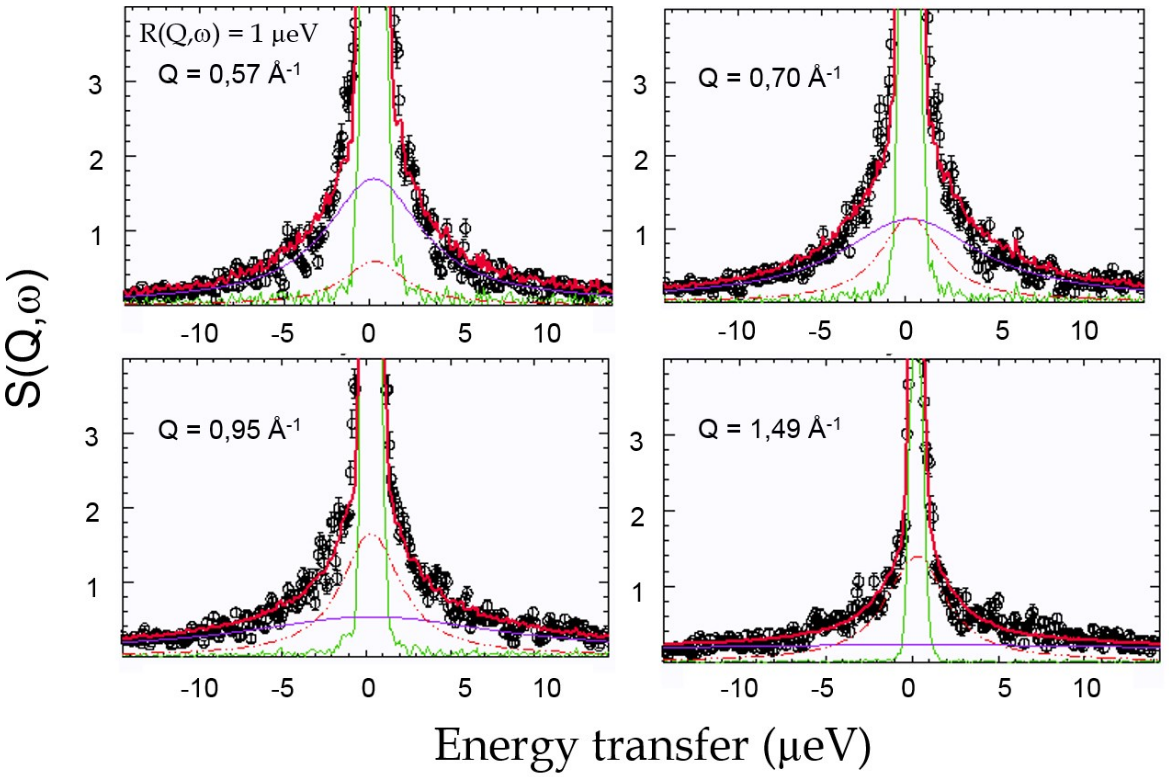

Figure S4. S(Q,ω) BS spectra of Aquivion membrane hydrated at RH = 22% measured on IN16 (at ILL). Q values are given in the figure. Data are fitted with Eq.1: fit (red line), $S_F(Q,\omega)$ (purple line), $S_S(Q,\omega)$ (Eq.2, dashed red line), P(Q) (green line).

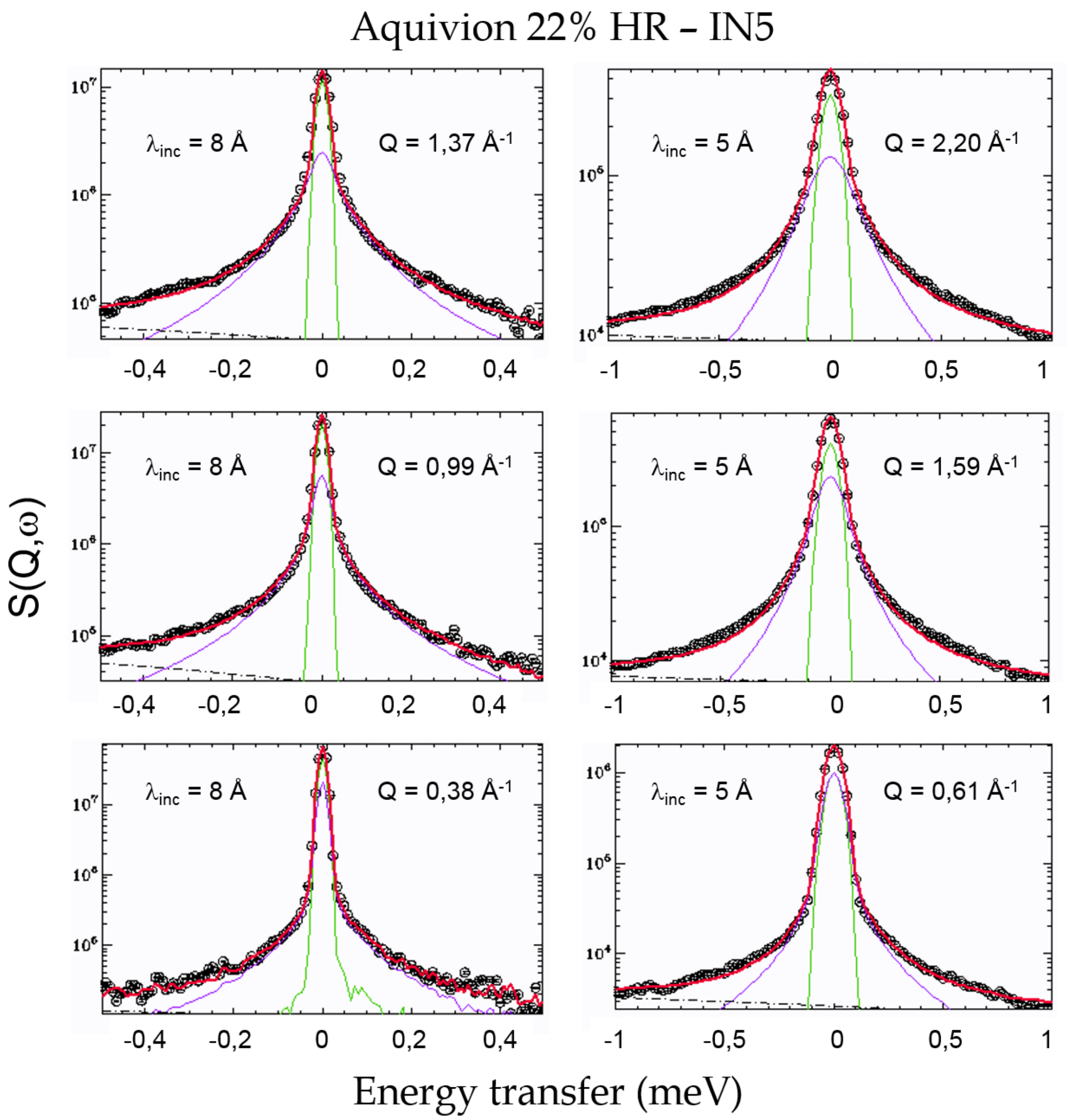

Figure S5. S(Q,ω) ToF spectra of Aquivion membrane hydrated at RH = 22% measured on IN5. Q values and incoming wavelength are given in the figure. Data are well reproduced over the Q-range using Eq.1 based on the generalized Gaussian model: fit (red line), $S_F(Q,\omega)$ (purple line), P(Q) (green line).

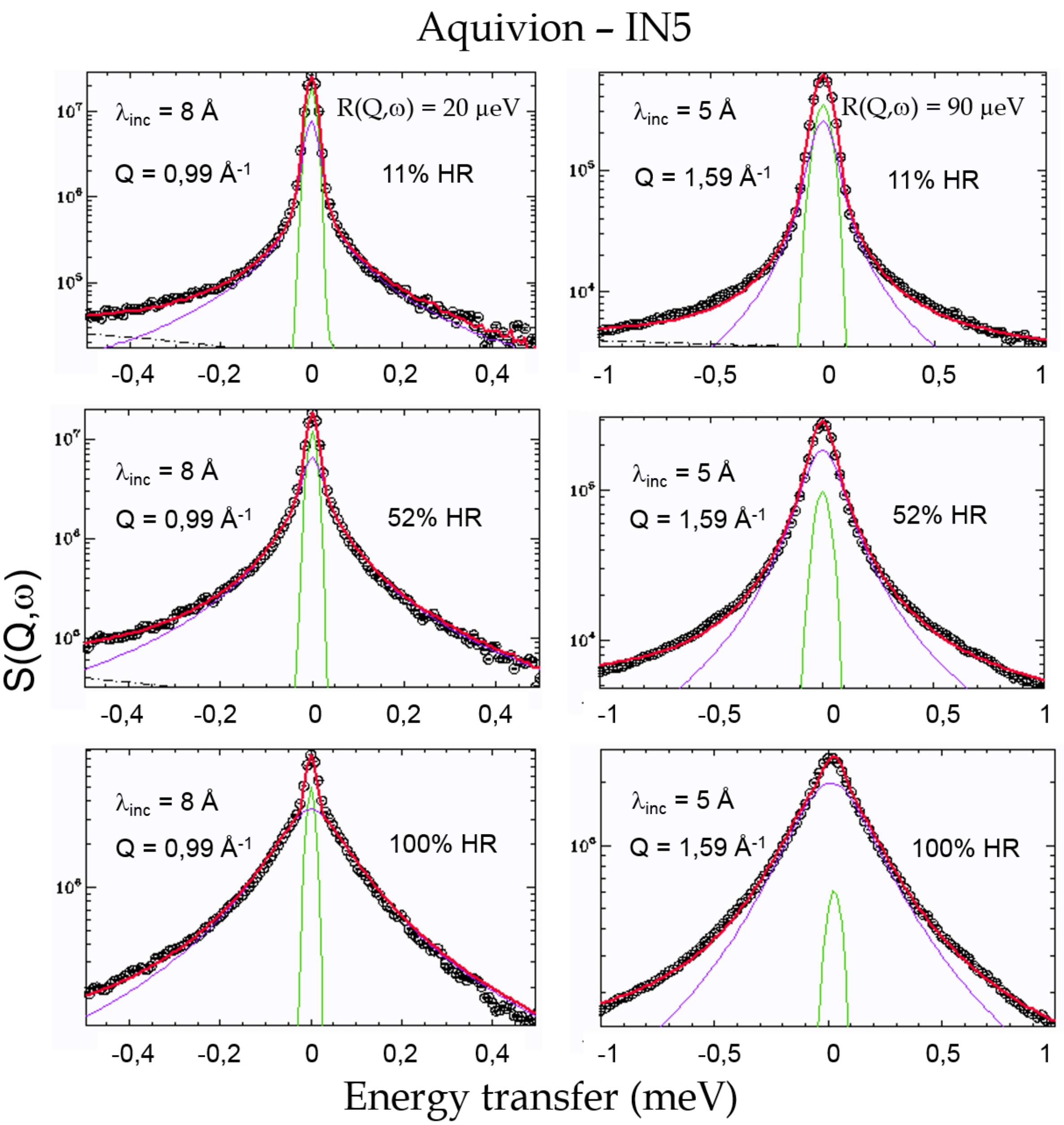

Figure S6. S(Q,ω) ToF spectra of Aquivion membrane hydrated at RH = 11%, 22% and 100 % RH, measured on IN5. Q values, RH and the incoming wavelength are given in the figure. Data are well reproduced over the hydration range using Eq.1 based on the generalized Gaussian model: fit (red line), $S_F(Q,\omega)$ (purple line), P(Q) (green line).

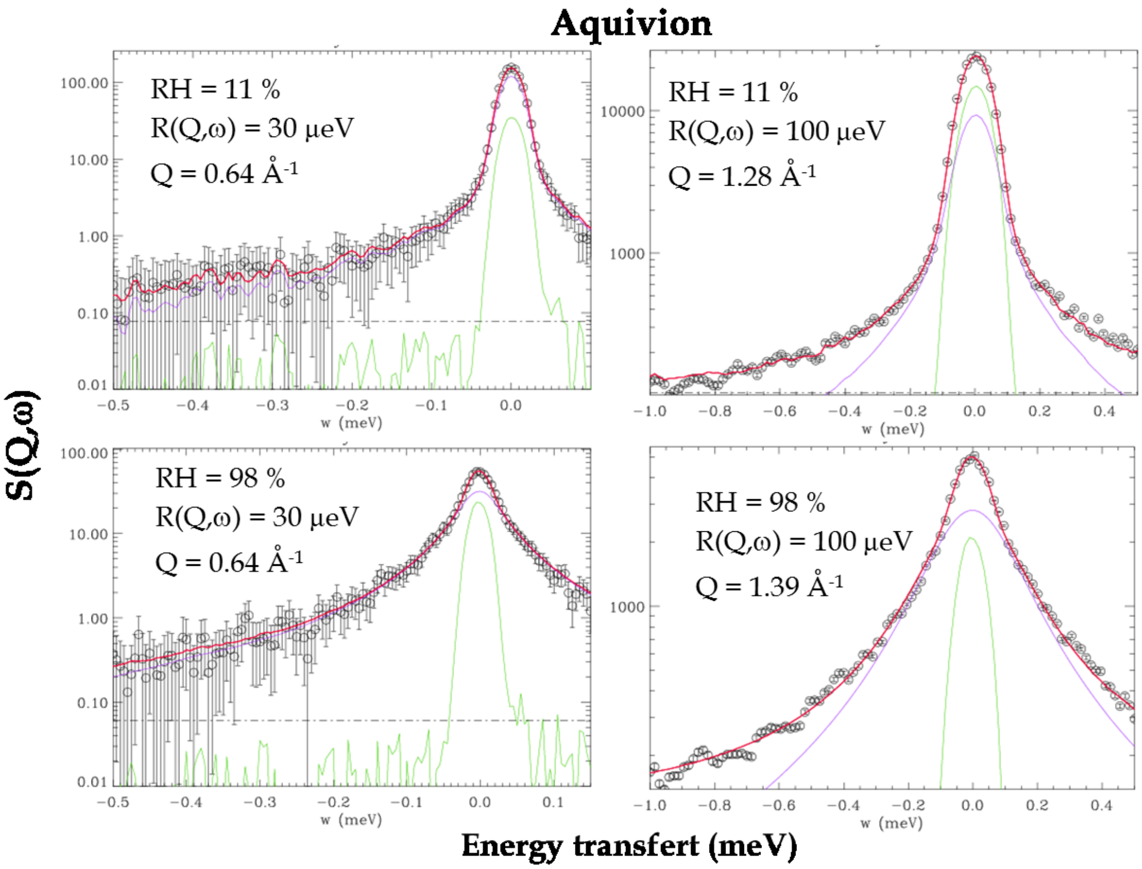

Figure S7. S(Q,ω) ToF spectra of Aquivion membrane hydrated at 11% and 98% RH, measured on Mibémol. Q values, RH and the instrumental resolutions are given in the figure. Data are well reproduced over the Q-range and the various hydrations using the generalized Gaussian model: fit (red line), $S_F(Q,\omega)$ (purple line), P(Q) (green line), B(Q) (black dashed line).

3.2. Parameters and their dependence upon hydration.

Values of the parameters: τ, τ_S, σ, D_{loc} and D_{nano} were extracted from the fit of the extended set of QENS spectra. N_F, N_S, and σ_S are extracted from the slow motions quasielastic structure factor analysis (see explanations below). All parameters are summarized in Tables S1 (slow dynamics) and S2 (fast dynamics), and plotted in Fig. S8-S10. Table S3 assembles the structural and dynamical parameters used in Fig. 4.

RH (%)	λ	$2\sigma_S$ (Å)	Γ_S (μeV)	τ_S (ps)	N_S	N_F
Dry	1.2	2.0 ± 0.2	1.5 ± 0.4	439 ± 117	3.4	-
11	3.7	2.70 ± 0.15	1.75 ± 0.3	376 ± 64	4.2	4.2
22	5.2	2.80 ± 0.14	2.0 ± 0.3	329 ± 49	5.2	6.2
52	5.8	3.20 ± 0.12	2.5 ± 0.3	263 ± 32	3.7	8.9
75	7.1	3.60 ± 0.12	3.5 ± 0.4	188 ± 21	3.4	11.8
85	8.3	4.00 ± 0.12	3.75 ± 0.5	175 ± 23	3	14.6
98	15.8	4.3 ± 0.2	4.0 ± 0.5	165 ± 21	3.7	28.9

Table S1. Aquivion slow dynamics parameters obtained from the fits with the generalized Gaussian model.

RH (%)	λ	2σ (Å)	D_{loc} (10^{-5} cm²/s)	τ (ps)	D_{nano} (10^{-5} cm²/s)
11	3.7	1.0 ± 0.5	0.40 ± 0.04	6 ± 1	0.10 ± 0.03
32	4.4	1.5 ± 0.5	0.65 ± 0.06	5.0 ± 0.5	0.15 ± 0.03
22	5.2	1.5 ± 0.6	0.65 ± 0.05	5.0 ± 0.5	0.15 ± 0.02
52	5.8	2.0 ± 0.4	0.90 ± 0.02	5.0 ± 0.5	0.20 ± 0.02
75	7.1	3.0 ± 0.2	0.90 ± 0.02	5.0 ± 0.5	0.30 ± 0.04
85	8.3	3.4 ± 0.3	0.95 ± 0.02	4.5 ± 0.4	0.33 ± 0.05
90	10.1	3.6 ± 0.3	1.00 ± 0.02	4.0 ± 0.3	0.35 ± 0.10
98	15.8	4.0 ± 0.3	1.20 ± 0.02	4.0 ± 0.3	0.5 ± 0.1
100	21.5	4.0 ± 0.3	1.5 ± 0.02	3.0 ± 0.3	0.5 ± 0.1

Table S2. Aquivion fast dynamics parameters obtained from the fits with the generalized Gaussian model.

λ	3.7		21.5		15.8		7.1		4.4	
**Aquivion**	l_{exp} (Å)	D (10^{-5} cm^2/s)	l_{exp} (Å)	D (10^{-5} cm^2/s)	l_{exp} (Å)	D (10^{-5} cm^2/s)	l_{exp} (Å)	D (10^{-5} cm^2/s)	l_{exp} (Å)	D (10^{-5} cm^2/s)
**loc**	1.00	0.40	4.00	1.50	4.00	1.00	3.00	0.90	1.50	0.65
**nano**	3.30	0.10	9.88	0.70	9.40	0.60	8.89	0.30	5.14	0.15
**µm**	22583	0.09	67082	0.75	63875	0.68	39497	0.26	26833	0.12

λ	17.50		3.30		4.70		7.90		10.00		3.90		5.90	
**Nafion**	l_{exp} (Å)	D (10^{-5} cm^2/s)	l_{exp} (Å)	D (10^{-5} cm^2/s)	l_{exp} (Å)	D (10^{-5} cm^2/s)	l_{exp} (Å)	D (10^{-5} cm^2/s)	l_{exp} (Å)	D (10^{-5} cm^2/s)	l_{exp} (Å)	D (10^{-5} cm^2/s)	l_{exp} (Å)	D (10^{-5} cm^2/s)
**loc**	4.20	1.50	2.10	0.45	2.40	0.70	3.60	1.00	4.10	1.00	2.20	0.50	2.80	0.90
**nano**	11.90	0.50	7.81	0.08	7.45	0.21	11.17	0.30	9.99	0.34	5.96	0.20	9.25	0.23
**µm**	54772	0.50	13416	0.03	21354	0.08	36332	0.22	43128	0.31	17321	0.05	28983	0.14

λ	1.50		3.10		4.90		18	
**PFOS**	l_{exp} (Å)	D (10^{-5} cm^2/s)	l_{exp} (Å)	D (10^{-5} cm^2/s)	l_{exp} (Å)	D (10^{-5} cm^2/s)	l_{exp} (Å)	D (10^{-5} cm^2/s)
**loc**	2.90	0.68	2.90	0.72	3.70	0.85	4.20	2.00
**nano**	12.40	0.07	8.10	0.27	11.20	0.27	12.20	0.70
**µm**	7746	0.01	24495	0.10	43128	0.31	64807	0.70

Table S3. Parameters used for Figure 4. Diffusion coefficients are given at the local (D_{loc}, extracted from QENS), nano (D_{nano}, extracted from QENS) and micrometric scale (D_S, measured by PFG-NMR). l_{exp} is the associated length scale. It corresponds to 2σ, 2L and $(6D_St)^{1/2}$, respectively.

The number of fast and slow protons, N_F and N_S, are reported in Fig. S8a together with those of Nafion membranes. N_F linearly increases with hydration, while N_S is hydration-independent, with a mean value of 3 protons per ionic groups. The characteristic times associated to each type of motions are shown on Fig. S8b for both Aquivion and Nafion.

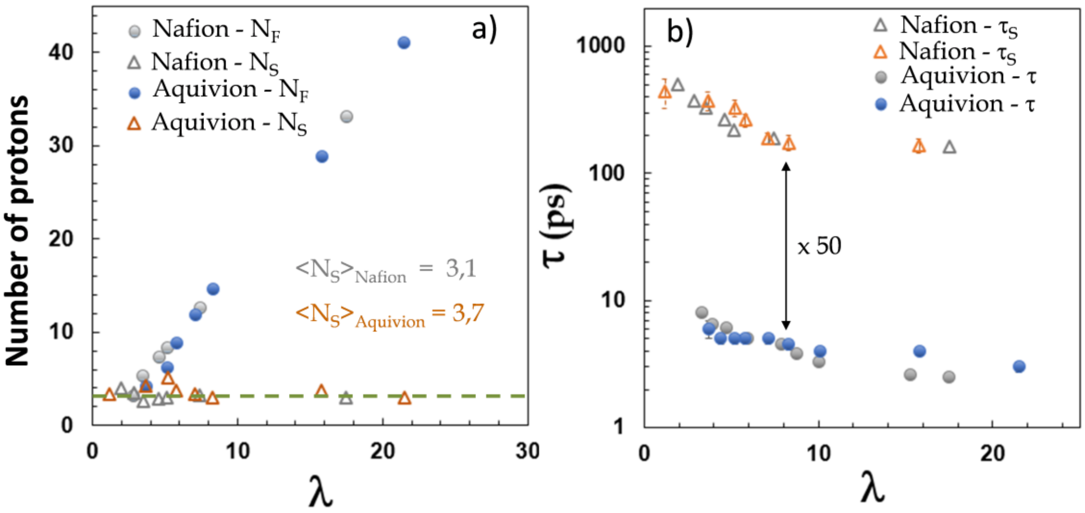

Figure S8. (a) Number of protons involved in the fast (N_F) and slow motions (N_S) as a function of the hydration number. (b) Characteristic times of fast (τ) and slow (τ_S) populations. Data are compared to Nafion (grey).[2]

In Fig. S9a, the evolution of the quasielastic intensity with hydration of the Aquivion's slow component $S_S(Q,\omega)$ is reported. $A_S(Q)$ is experimentally determined from the ratio of the two quasielastic component integrals (the slow component / the fast one). With this method, the Debye-Waller factor is eliminated, and we directly obtain the quantity $A_S(Q) \times N_S/N_F$. The latter is fitted with the Gaussian model EISF:[3] $A_S(Q) = 1 - \exp(-Q^2\sigma_S^2)$ where $2\sigma_S$ is the jump distance (see Fig. S3). τ_S, the residence time is determine with the backscattering QENS measurements (IN16, Fig. S4). The ratio N_S/N_F is determined from the previous fit, and each value are obtained from the relation $N_F + N_S = 2\lambda+1$. The evolution of τ_S and $2\sigma_S$ with the hydration is shown in Fig. S9b,c, for Nafion,[2] PFOS,[4] and Aquivion.

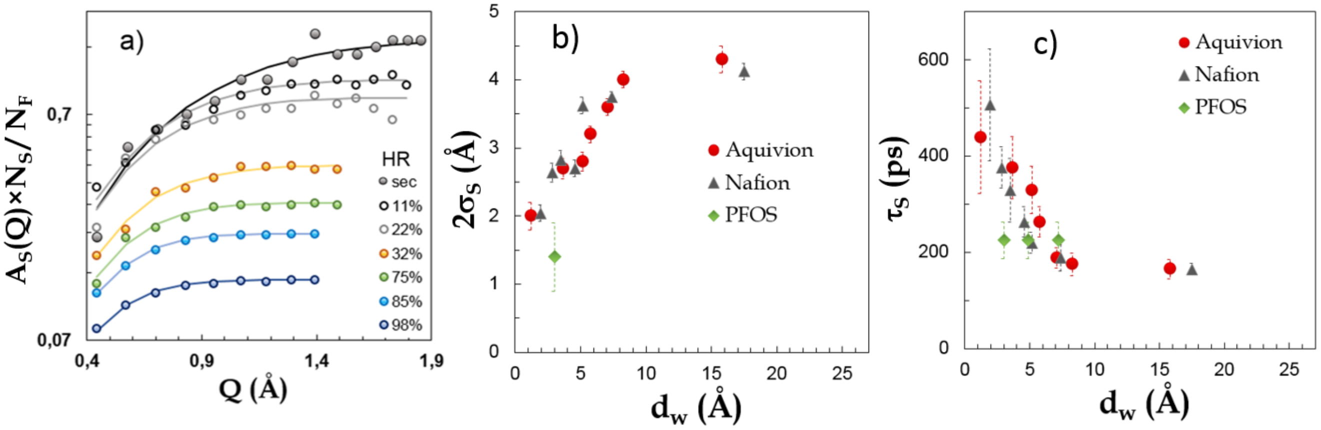

Figure S9. Evolution with the hydration of a) the structure factor $A_S(Q)\times N_S / N_F$, and b) the jump distance $2\sigma_S$, and c) the residence time τ_S. Data are compared to Nafion[2], and PFOS.[4]

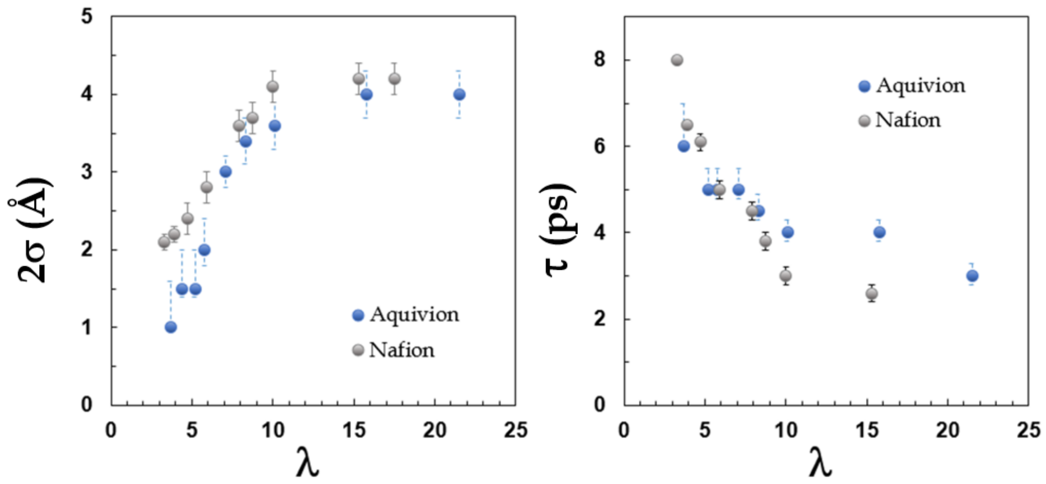

Figure S10. Confinement sizes 2σ (left) and characteristic relaxation times τ (right) versus λ obtained for the fast protons of the Aquivion membrane. Data are compared to Nafion.[2]

4 – Self-diffusion coefficient of water by PFG-NMR.

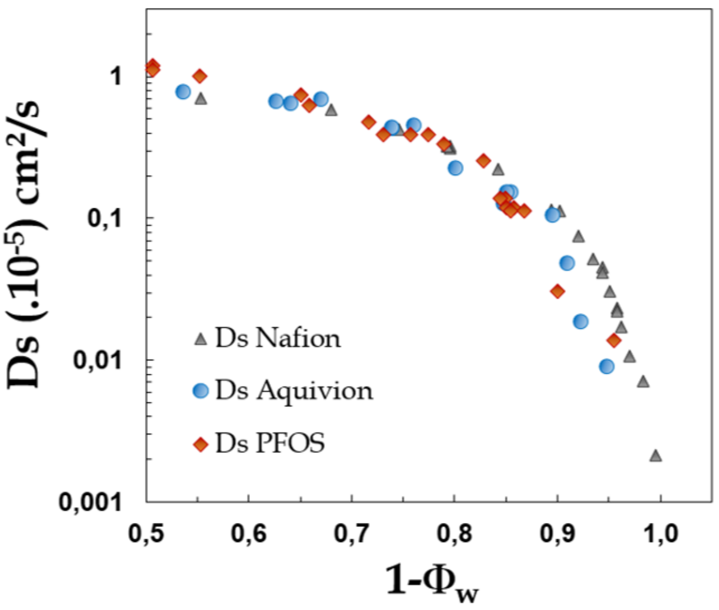

Figure S11. Aquivion self-diffusion coefficient of water, D_S, determined by PFG-NMR and plotted versus 1-Φ_w. The values in Aquivion are compared to those found in Nafion and PFOS.[1] The behavior at large water volume fraction is found to be independent of the details of chemical architecture, as due to the driving “obstacle mechanism”, e.g. water molecules diffuse within large water pools embedding hydrophobic aggregates.

5 – MD simulations.

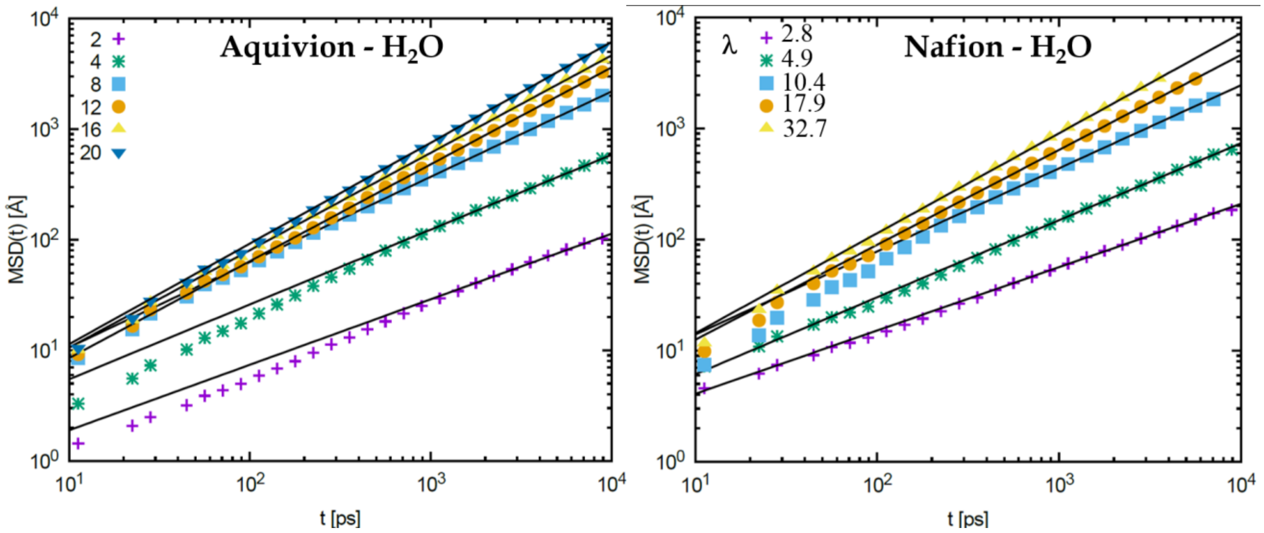

Figure S12. MSD(t) of water molecules in Aquivion and Nafion as a function of the hydration. MSD(t) are calculated as described in ref[5].

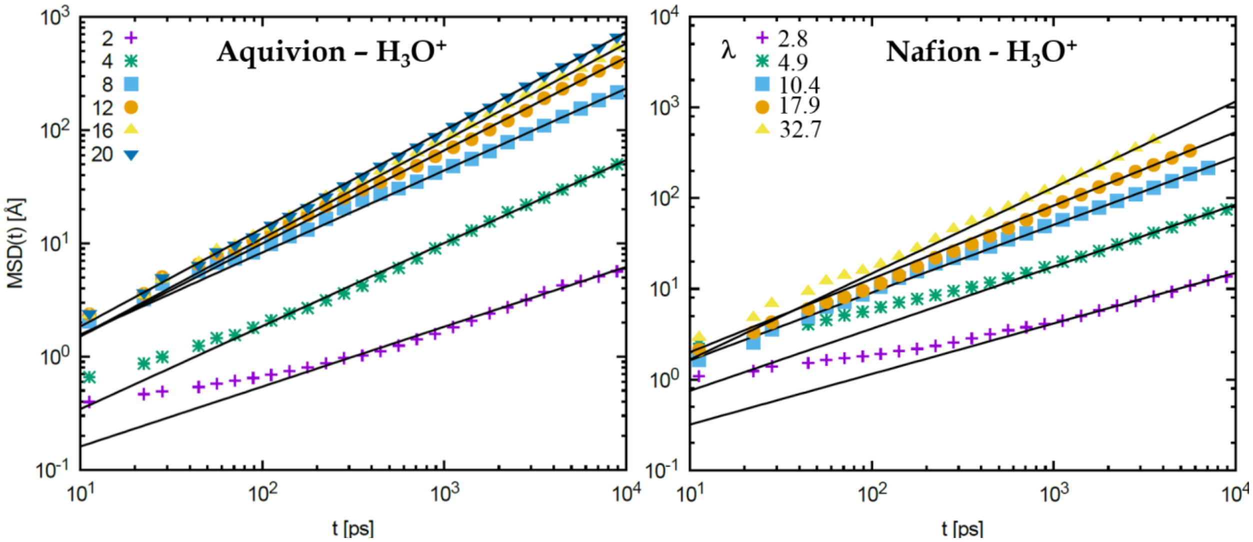

Figure S13. MSD(t) of hydronium ions in Aquivion and Nafion as a function of the hydration. MSD(t) are calculated as described in ref[5].

Material	Model	Fast component (Fig. 4-5)	Slow localized jumps (Fig. 6)
Nafion[2]	GMLTM[6]	Localized diffusion: σ, D_{loc}, τ	$2\sigma_s$, τ_s
Aquivion (this work)	GMLTM[6]	Fickian diffusion: D_{nano}	$2\sigma_s$, $\tau_{s,}$ D_{slow}
Aquivion[4]	Multi-lorentzian	Confined random jump diffusion: σ, D_{loc}, τ	τ_s
PFOS[7]	GMLTM	σ, D_{loc}, τ, D_{nano}	-
PFOS[4]	Multi-lorentzian	Confined random jump diffusion: σ, D_{loc}, τ	τ_s

Table S4. Models used in this work and previous publications.

REFRENCES:

1 Q. Berrod, S. Lyonnard, A. Guillermo, J. Ollivier, B. Frick, A. Manseri, B. Améduri and G. Gébel, *Macromolecules*, 2015, **48**, 6166–6176.

2 J.-C. Perrin, S. Lyonnard and F. Volino, *J. Phys. Chem. C*, 2007, **111**, 3393–3404.

3 F. Volino, J. C. Perrin and S. Lyonnard, *J. Phys. Chem. B*, 2006, **110**, 11217–11223.

4 Q. Berrod, S. Lyonnard, A. Guillermo, J. Ollivier, B. Frick and G. Gébel, *EPJ Web Conf.*, 2015, **83**.

5 S. Hanot, S. Lyonnard and S. Mossa, *Nanoscale*, 2015, 3314–3325.

6 F. Volino, J. C. Perrin and S. Lyonnard, *J. Phys. Chem. B*, 2006, **110**, 11217–11223.

7 S. Lyonnard, Q. Berrod, B.-A. Brüning, G. Gebel, A. Guillermo, H. Ftouni, J. Ollivier and B. Frick, *Eur. Phys. J. Spec. Top.*, 2010, **189**, 205–216.
